# Supplementary material for: Association between oral microbiome and seven types of cancers in East Asian population: a two-sample Mendelian randomization analysis
Source: Front Mol Biosci. 2023 Nov 21;10:1327893. doi: 10.3389/fmolb.2023.1327893 (PMC10702768; doi:10.3389/fmolb.2023.1327893)
Supplement: Supplementary file 1 [file DataSheet1.ZIP › Supplementary Materials/MR plots for tongue/tongue═╝/Colorectal cancer/pheno.982_to_colorectal cancer_forest.pdf]

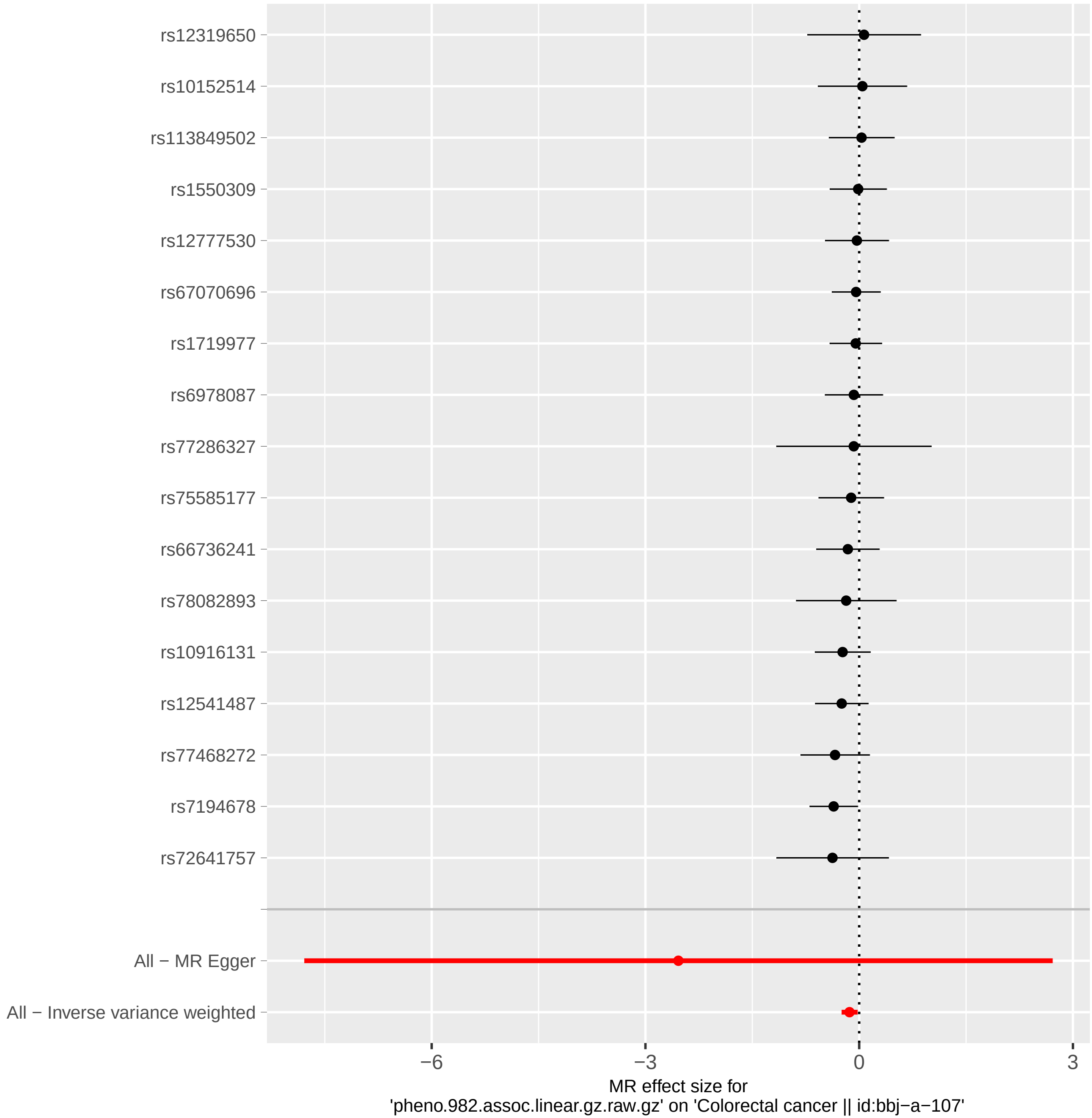
rs12319650
rs10152514
rs113849502
rs1550309
rs12777530
rs67070696
rs1719977
rs6978087
rs77286327
rs75585177
rs66736241
rs78082893
rs10916131
rs12541487
rs77468272
rs7194678
rs72641757
All − MR Egger
All − Inverse variance weighted
−6
−3
0
3
MR effect size for
'pheno.982.assoc.linear.gz.raw.gz' on 'Colorectal cancer || id:bbj−a−107'
